# Parental risk perceptions of child exposure to tobacco smoke

**DOI:** 10.1186/s12889-015-1434-x

**Published:** 2015-02-06

**Authors:** Laura Rosen, Inessa Kostjukovsky

**Affiliations:** Department of Health Promotion, School of Public Health, Sackler Faculty of Medicine, Tel Aviv University, Ramat Aviv, Israel; Netanya Regional Health Bureau, Ministry of Health, Netanya, Israel

**Keywords:** Tobacco smoke exposure (TSE), Secondhand smoke exposure (SHS, SHSe), Passive smoking, Risk perceptions, Infants, Pediatric, Smoke-free homes

## Abstract

**Background:**

Tobacco smoke exposure harms children and adults. Yet, 40% of children worldwide are exposed to tobacco smoke in their homes. Such widespread parental failure to protect children is puzzling, and may be related to risk perceptions. No consensus exists about how to measure parental risk perceptions of tobacco smoke exposure.

**Methods:**

The objective of this research was to study **P**arental **R**isk Perceptions of child **E**xposure to **T**obacco **S**moke (PRETS) using various dimensions of risk perceptions: likelihood of harm, susceptibility to harm, and severity of harm. We aimed to estimate PRETS and identify correlates of PRETS, and assess the association between PRETS, parental smoking status, and home smoking behaviors. We conducted 132 face-to-face interviews with parents of infants.

**Results:**

Parents who smoked regularly believed that infant tobacco smoke exposure was less dangerous than did other parents (p = .0158). Birthplace of parent was significantly associated with risk perception (p = .0019); parents of Russian origin believed the overall risk to be less than did those born elsewhere. Smoking status, ethnicity, and employment status were associated with smoking in the home. The relationship between smoking behavior in the home and risk perceptions was complex, and may have been modified by ethnicity.

**Conclusions:**

Parental risk perceptions concerning child exposure to tobacco smoke are associated with smoking behavior and ethnicity. Understanding how to measure risk perceptions, and identifying risk perception dimensions which differ between families with and without home smoking bans, may contribute to the development of effective interventions to protect children from the harmful effects of tobacco smoke exposure.

## Background

Despite the accumulated evidence of harm to children from tobacco smoke exposure (TSE), [1,2] roughly 40% of children worldwide are exposed to tobacco smoke in their homes [3]. Infants are perhaps the most vulnerable to harm: captive smokers in their own homes, with their small bodies and developing lungs, they are at increased risk of sudden infant death syndrome, acute respiratory infections, lower respiratory illness, acute and recurrent otitis media and chronic middle ear effusion, onset of wheeze illnesses, cough, phlegm, wheeze, breathlessness, asthma diagnosis, continued adverse effects on lung function, and delayed lung maturation [1].

The primary source of young child exposure and of exposure-related harm to the child is smoking in the child’s home [4]. The phenomenon of parents either causing harm to their own children by smoking around them in their homes, or of allowing others to harm them by smoking in their presence, is inherently puzzling. One possible explanation for this phenomenon is that parents underestimate risks associated with child TSE, and therefore don’t protect them. This would be consistent with the approach of the Health Belief Model (HBM), which posits that “the perceptual world of the behaving individual” is related to preventive health behaviors. In the HBM formulation, susceptibility to harm and severity of harm are two critical dimensions of health perceptions [5]. These dimensions are thought to be at least partially dependent on knowledge [5]. More recently, a third dimension, likelihood of harm, has been identified [6]. Risk perceptions regarding various types of health behaviors -- for example vaccinations, fruit intake, dental checkups, and drinking and driving -- have been addressed by researchers using some of these dimensions [6,7]. Though research has been conducted on the relationship between risk perceptions, knowledge, and passive smoking, [8-17] research exploring dimensions of risk perceptions [6] has not, to the best of our knowledge, been conducted for the field of child TSE.

The goals of this study were to 1 - pilot a method for measuring Parental Risk perceptions of Exposure to Tobacco Smoke (PRETS), using established dimensions of risk perception; 2- identify correlates of PRETS, and 3- explore the relationship between PRETS, parental smoking status, and family smoking in the home. We hypothesized that PRETS were associated with parental smoking status and family smoking in the home.

## Methods

### Study design and recruitment strategy

The study was a face-to-face survey of parents who visited a large well-baby clinic (Tipat Chalav) in central Israel in order to vaccinate their infants in 2008. All parents of infants up to the age of 14 months were potentially eligible to participate in the survey.

We attempted to recruit an equal number of regular smokers and others, during approximately the same time period. It was important to interview parents with different smoking behaviors concurrently because at the time of the study, a new law for preventing smoking in public places had recently been passed, resulting in broad media coverage and a lively public discourse on the dangers of secondhand smoke exposure [18]. As there were fewer regular smokers than others in the population, simply recruiting on a first-come-first-entered basis could have served to introduce a bias, as those recruited earlier may have had less exposure to the public discussion than those recruited later. We used the following strategy to ensure that regular smokers and others were recruited during the same time period: Whenever a potential participant arrived at the clinic for a regular appointment, the nurse/secretary asked about his/her smoking habits. If the person was a regular smoker, s/he was invited to join the study. Each time a regular smoker was recruited, the next non-regular smoker to arrive at the clinic was recruited for the study. After that non-regular smoker was recruited, a regular smoker was recruited.

### Questionnaire development

We were interested in measuring risk perceptions of parents regarding child exposure to tobacco smoke and knowledge regarding child exposure to tobacco smoke, smoking practices in the home and car, smoking status of respondents, and demographic and socio-economic variables. We built a questionnaire which was influenced by previous work, particularly the work of Brewer [6], Johansson, [19] Bock, [9] and the Global Youth Tobacco Survey [20].

### Risk perceptions

We constructed three questions, each corresponding to a dimension of risk perception as defined by Brewer: perceived likelihood, perceived susceptibility, and perceived severity [6]. For perceived likelihood (“the probability that one will be harmed by the hazard”) our question was: “In your opinion, is it reasonable that a child exposed to secondhand smoke will get a respiratory tract infection?”. For perceived susceptibility (“An individual's constitutional vulnerability to a hazard”) the question was: “In your opinion, is a child exposed to SHS more likely to get a respiratory tract infection than other children?” For perceived harm, (“The extent of harm a hazard would cause”), the question asked was: “In your opinion, how much will tobacco smoke in the child’s environment affect your child’s health?” Severity has also been defined as concerns about clinical or social consequences [7].

We note that our definition of susceptibility differs from the ones used by Brewer [6] or by Janz [7]. In our framework, susceptibility is by definition due to tobacco smoke exposure, and not due to individual constitutional vulnerability. The difference between the dimensions of likelihood and susceptibility, as defined here, is that likelihood refers to whether someone exposed to tobacco smoke is likely to have an untoward event occur, whereas susceptibility is a comparative question, and asks whether an untoward event is more likely to occur to someone exposed to tobacco smoke than to someone unexposed.

We further note that some previous investigators have chosen to phrase questions in personal terms, while others have chosen to phrase them in general terms. For example, Brewer 2007 [6] reported that for assessing severity, Zimmerman et al. used a personal question: “If I had influenza, I would not be able to manage daily activities” while Nichol et al. 1992 used the impersonal question: “Influenza can cause death.” In our study, likelihood and susceptibility were asked in general terms, about children exposed to tobacco smoke, while severity was asked in personal terms.

The questions relating to the dimensions of risk perception are presented in Table 1. All questions were asked using a 1–7 Likert scale. Seven point scales have been recommended for risk perception questions [21]. Responses to the three questions were summed to create an overall scale for risk perception.

**Table 1:** **Dimensions of risk perception**

**Dimension**	**Description of dimension by Brewer**	**Tobacco smoke exposure question**
Perceived likelihood	The probability that one will be harmed by the hazard	In your opinion, is it reasonable that a child exposed to secondhand smoke will get a respiratory tract infection? (1 = completely unreasonable, 7 = absolutely)
Perceived susceptibility	An individual's constitutional vulnerability to a hazard	In your opinion, is a child exposed to secondhand smoke more likely to get a respiratory tract infection than other children? (1 = Not at all susceptible, 7- Most susceptible)
Perceived severity	The extent of harm a hazard would cause	In your opinion, how much will tobacco smoke in the child’s environment affect your child’s health? (1 = No influence, 7 = Very strong influence)

### Knowledge questions

We also asked about knowledge regarding harm due to TSE. We constructed a scale for knowledge from the five questions about knowledge due to harm to children from tobacco smoke exposure. To create the scale, we reverse-coded Question 23 and then summed the responses.

### Smoking behavior

‘Regular smokers’ included those who smoked daily or almost daily, while ‘others’ included occasional smokers (less than almost daily), past smokers, and never smokers.

We chose to classify smoking behavior into these two categories based on results from a pretest of these questions, at which time we saw that the biggest differences in perceptions were between regular smokers and others. Previous researchers have used various categorizations: Drehmer [22] categorized smoking behavior by < =10 vs. >10 daily cigarettes; Chen 2013 [10] defined smokers as those who smoked at least 100 cigarettes during their lifetime and at least one in past 30 days; and Wagener 2010 [23] defined smokers as those who smoked > =3 cigarettes per day for the past year.

Respondents were asked whether they or family members usually smoked at home (yes/no) or in the car (yes/no).

### Additional variables

Additional potential explanatory variables included: age, gender, years of education, work status (full-time, part-time, or unemployed), country of birth of the parent, family income, marital status (single, married, divorced, widowed), religiosity (Secular, traditional, religious, Ultra-Orthodox), number of children, age of youngest child, and length of lactation (<1 month, 1–3 months, 3–6 months, >6 months).

### Validation of instrument

We ran several pretests of the questionnaire, with participants recruited from a different clinic. The first pretest included 64 participants [24]. Problematic questions were revised. We ran an additional pretest with 10 individuals using a test/retest approach, at an interval of 3–5 weeks. The Pearson correlation coefficients for the test/retest were: Likelihood, r = .55; Susceptibility, r = .53; Severity: r = .75. Full details are reported elsewhere [24].

### Statistical analyses

We conducted the following analyses:

1. Smoking status: We compared regular smokers and others on socio-demographic variables. T-tests were used to compare continuous variables and Chi-squared tests were used to compare categorical variables.
2. Risk perception and knowledge: We calculated Pearson correlation coefficients to study the association between risk perception and knowledge questions and scales, as well as Cronbach’s alpha for the risk perceptions and knowledge scales. We compared each of the individual questions and the combined scales by smoking status, using t-tests (as has been done or recommended by other authors when using for a 7-point Likert scale [21,25,26]).
3. Family smoking in the home and car: We compared smoking behavior in the home and car, and smoking status, using chi-squared statistics.
4. Statistical models: We examined a. the relationship between PRETS and smoking status, by using multivariate analysis of variance, with PRETS defined as the outcome variable, and smoking status and socio-demographic as explanatory variables; b. the relationship between family smoking in the home and PRETS, using multiple logistic regression, with family smoking in the home defined as the binary outcome variable, and risk perception and parental smoking status as explanatory variables; c. the relationship between family smoking in the home and PRETS, by using multiple logistic regression, with family smoking in the home defined as the binary outcome variable, and risk perception, parental smoking status, and socio-demographic explanatory variables, and d. an exploratory analysis similar to “c” in which we excluded ethnicity from the model.

SAS 9.2 was used for the statistical analysis, and SPSS 21 was used to create the graphs.

### Ethical approval

The study was approved by the Tel Aviv University Ethics Committee, by the Central District Health Bureau, and by the Netanya Regional Health Bureau officials. Written informed consent was fobtained from all participants.

## Results

### Participants

We approached 135 potential participants, of whom 132 (97.8%) agreed to participate.

Most participants (80.3%) were mothers, with the mean age of around 30. Sixty (45.5%) were daily smokers, 6 were almost-daily smokers (4.5%), 11 (8.3%) were occasional smokers, 12 (9.1%) were past smokers, 10 (7.6%) were nonsmokers who had at some point experimented with smoking, and 33 (25%) were never smokers. Comparisons between regular smokers and others on socio-demographic and other variables are shown in Table 2. The following significant differences were found: Fathers, single or divorced parents, and secular or traditional parents were more likely to be regular smokers. Regular smokers had fewer years of education than did others.

**Table 2:** **Descriptive information on participants, by smoking status**

**Variable**	**Entry**	**Others (N = 66)**	**Regular smoker (N = 66)**	**p-value for difference between groups**
Age (in years)	Mean (STD)	30.0 (5.1)	29.3 (5.6)	.4367
Years of education	Mean (STD)	14.4 (2.1)	12.7 (1.9)	<.0001
Number children	Mean (STD)	2.1 (1.2)	1.7 (1.2)	.0887
Religiosity	Secular/Traditional	43.8%	56.2%	.0050
	Religious/Ultra-Orthodox	74.1%	25.9%	
Parent	Mother	58.5%	41.5%	<.0001
	Father	15.4%	84.6%	
Family financial status (New Israeli Shekels, per month)	<5000	31.6%	68.4%	.2262
	5000-12000	51.8%	48.2%	
	12,000+	54.8%	45.2%	
Origin	Israel	44.0%	56.0%	.3078
	Russia	50.0%	50.0%	
	Other	63.6%	36.4%	
Marital status	Single	16.7%	83.3%	.0092
	Married	54.7%	45.3%	
	Divorced	0.0%	100.0%	
Work	Full-Time	45.0%	55.0%	.2751
	Part-Time	64.0%	36.0%	
	None	48.9%	51.1%	
Number of cigarettes per day	Mean (STD)	1.1 (0.35)	5.2 (0.90)	<.0001
Family members smoke at home (% yes)		16.7%	78.8%	<.0001
Family members smoke in car (% yes)		15.2%	50.0%	<.0001
Lactate	0-3 months	47.1%	52.9%	.0014
	4+ months	79.0%	21.0%	

### Risk perceptions and knowledge

Correlations between the three dimensions of risk perception were statistically significant (p < .0001). The correlations were .64 for likelihood and susceptibility, .60 for likelihood and severity, and .75 between susceptibility and severity. Cronbach’s alpha for the three components – likelihood, susceptibility, and severity -- was .85.

Table 3 presents descriptive statistics and comparisons for the three components of risk perception and the combined score. Regular smokers were less aware of risk than were others (Combined score: Regular Smokers: Mean:14.03, Standard Deviation (STD):4.67, Others: Mean: 16.65, STD: 3.39. Differences between regular smokers and others were significantly different for all three dimensions of risk (Likelihood p-value:.0440 Susceptibility p-value:.0002 Severity p-value:.0001), and the combined score (p = .0003). Among the three dimensions, the biggest absolute difference, and the most extreme p-value, occurred for the severity dimension.

**Table 3:** **Risk perceptions and knowledge by smoking status and by exposure in home**

**Mean (STD)**	**Others (N=66)**	**Regular smokers (N=66)**	**p-value** ***t*** **-test**	**Family does not smoke in home (N=69)**	**Family smokes in home (N=63)**	**OR [CI] p-value (logistic regression)**
Q19. **Likelihood**. Is it reasonable that a child exposed to passive smoking will be sick with respiratory illnesses? (1=Completely unreasonable 7- Absolutely)	5.14 (1.41)	4.53 (1.96)	.0440	5.19 (1.53)	4.44 (1.86)	.81 [.62,1.06] .1228
Q20. **Susceptbilty**. Will an infant who is exposed to passive smoking be more susceptible to respiratory illness than other children? (1=Not at all susceptible 7- the most susceptible)	5.67 (1.22)	4.68 (1.72)	.0002	5.68 (1.29)	4.62 (1.66)	.71 [.52,.97] .0319
Q21. **Severity**. How much can exposure to smoke around your child influence his health? (1=No influence 7- Very strong influence)	5.85 (1.19)	4.82 (1.73)	.0001	5.83 (1.12)	4.79 (1.80)	.75 [.55,1.01] .0595
**Combined risk perception**	16.65 (3.39)	14.03 (4.67)	.0003	16.7 (3.33)	13.86 (4.71)	.89 [.79,.88] .0350
Q23. Childhood illnesses are not associated at all with smoking around the child (1=Completely disagree, 7=Agree a lot)	3.64 (1.89)	4.79 (1.92)	.0007	4.01 (1.95)	4.43 (2.01)	1.15 [0.91,1.47] .2440
Q24. In my opinion, there is a link between the health of children and between smoking by their parents (1=Completely disagree, 7=Agree a lot)	6.11 (1.44)	4.52 (1.94)	<.0001	5.86 (1.63)	4.71 (1.96)	1.09 [0.84,1.40] .5215
Q25. In my opinion, passive smoking harms child development (1=Completely disagree, 7=Agree a lot)	5.27 (1.79)	4.03 (2.17)	.0005	5.30 (1.83)	3.94 (2.11)	1.28 [1.02,1.59] .0322
Q29. Breathing tobacco smoke is a risk factor for many diseases in infants (1=Completely disagree, 7=Agree a lot)	6.06 (1.18)	5.33 (1.69)	.0048	6.06 (1.27)	5.30 (1.62)	1.28 [0.93,1.75] .1326
Q30. Breathing tobacco smoke is a risk factor for child mortality (1=Completely disagree, 7=Agree a lot)	4.05 (1.84)	3.48 (2.09)	.1048.	4.13 (1.93)	3.37 (1.98)	1.20 [0.95,1.50] .1250
**Combined knowledge scale** (Q23 reversed, Q24, Q25, Q29, Q30) (Scale: 5–35)	25.85 (5.35)	20.58 (6.83)	<.0001	23.62 (3.3)	20.3 (5.14)	1.16 [1.03,1.31] .0124

Descriptive statistics regarding knowledge are also presented in Table 3. The combined knowledge construct had a Cronbach’s alpha of .75. Parents who smoked regularly scored lower on knowledge for each individual question (including q23 once it was transposed) and for the combined measure. The differences between regular smokers and others were statistically significant for the questions on child disease and development, but not for the question about child mortality.

The associations between the combined scores for risk perceptions and knowledge were statistically significant for both regular smokers (r = .538, p < .0001) and others (r = .465, p < .0001).

### Smoking behavior in home and car

Most (78.8%) regular smokers, and a minority (16.7%) of others, reported that they or family members usually smoked in their homes. Half (50.0%) of regular smokers, and a minority of others (15.2%) reported that they or their family members usually smoked in cars. Most families of regular smokers (57/66 = 86.4%) smoked in the home, car, or both. A minority of families of others (14/66 = 21.2%) smoked in the home, car, or both.

### Statistical models

Full results from statistical models 1, 3, and 4 are presented in Table 4. Model 1 shows the results of the statistical analysis of the effects of smoking status and other variables with risk perceptions: The R-squared for the multiple regression, with the combined risk perception scale as the response variable, was .24. Regular smokers evaluated risk perceptions as lower than did others (LSMeans, Regular smokers: 14.01, Others: 16.04, p = .0158). Ethnicity was statistically significant, with parents of Russian origin showing lower risk perceptions than others (LSMeans Russians: 13.26, Others: 15.54, Israeli: 16.29, p = .0019). None of the other variables reached statistical significance.

**Table 4:** **Statistical model results: risk perceptions and family smoking in the home**

	**Model 1: outcome: risk perception**	**Model 3: outcome: family smoking in home**	**Model 4: outcome: family smoking in home**
	**(N=131)**	**(N=131)**	**(N=131)**
	**Least squared means**	**Odds ratio**	**Odds ratio**
	**Type III p-values**	**[Confidence Interval],**	**[Confidence Interval],**
		**p-value**	**p-value**
Status (regular smoker vs. other)	Regular smokers: 14.01	Regular smoker vs. other:	Regular smoker vs. other:
	Others: 16.04	OR:45.00 [9.98,202.94]	OR:24.25 [6.90,85.20]
	p-value: .0158	p-value: <.0001	p-value: <.0001
Risk perception		OR: 0.93 [0.81,1.07]	OR: 0.88 [0.78,1.003]
		p-value: .3224	p-value: .0560
Parent (father vs. mother)	Father: 14.37	OR: 1.36 [0.30,6.15]	OR: 1.37 [0.34,5.59]
	Mother: 15.68	p-value:.6865	p-value:.6624
	p-value: .1974		
Work status	Full: 15.23	No vs.full-time: OR: 4.45	No vs.full-time: OR: 5.87
No vs. full-time	No: 14.93	[1.16,17.02]	[1.60,21.63]
Part vs. full-time	Part: 14.91	Part vs. full-time: OR: 0.44	Part vs. full-time: OR: 0.46
	p-value: .9214	[0.95,2.00]	[0.11,1.94]
		p-value:.0181	p-value:.0042
Origin	Israel: 16.29	Russia vs. Israel: OR:	Not in model
Russia vs. Israel	Other: 15.54	4.03[0.93,17.37]	
Other vs. Israel	Russia: 13.26	Other vs. Israel: 0.41	
	p-value: .0019	[0.09,1.96]	
		p-value: .0433	
Age (30 years + vs. <30 years)	30+: 14.42	30+ vs. <30: OR:1.09	30+ vs. <30: OR:0.75
	<30: 15.63	[0.34,3.52]	[0.26,2.20]
	p-value: .1192	p-value:.8885	p-value:.6056
Education (<=12 years vs.12+ years)	0-12:14.39	<=12 vs. 12+: 0.996 [0.32,3.07]	<=12 vs. 12+: 1.12 [0.39,3.25]
	>12:15.66	p-value:.9941	p-value:.8367
	p-value: .1145		
Religiosity (Ultra Orthodox and religious vs. secular or traditional)	Religious, Haredi:15.29	Haredi/religious vs.	Haredi/religious vs.
	Secular, traditional: 14.76	secular/traditional: 2.60	secular/traditional: 0.98
	p-value: .5977	[0.50,13.46]	[0.26,3.75]
		p-value:.2562	p-value:.9750
Marital	Divorced:12.83	Divorced vs. Married: 0.21	Divorced vs. Married: 0.12
(Divorced vs. Married	Married: 15.37	[0.01,7.57]	[0.004,3.08]
Single vs. Married)	Single: 16.87	Single vs. Married: 0.87	Single vs. Married: 1.47
	p-value: .2597	[0.08,8.99]	[0.18,11.90]
		p-value:.6875	p-value:.3205
Income (in thousands of New Israeli Shekels)	12+: 15.16	5-12 vs. 12+: 1.25 [0.36,4.42]	5-12 vs. 12+: 1.11[0.34,3.62]
	5-12: 14.57	5 vs. 12+: 1.04 [0.10,10.60]	5 vs. 12+: 0.76 [0.08,7.14]
5-12 vs. 12+	<5:15.35	p-value:.9278	p-value: .9221
<5 vs. 12+	p-value:.6644		

The variable, Risk Perception, was obtained by summing responses to three questions pertaining to individual dimensions of risk perception: likelihood, severity, and susceptibility. As those questions were asked on a scale from 1–7, the outcome variable can range from 3–21. Higher values indicate higher perceptions of risk.

Model 1: Multivariate analysis of variance. Models 3, 4: Multivariate logistic regression.

Models 2, 3, and 4 concern the relationship between risk perceptions and family smoking in home, while controlling for different sets of variables. In Model 2, just two covariates were included: combined risk perception, and parental smoking status. Parents who smoked regularly, and respondents with lower levels of risk perceptions, were more likely to live in families where smoking took place in the home (Regular smokers vs. Others: OR = 15.63, CI: [6.40,38.15], p < .0001), Risk perceptions: OR = 0.89,CI: [0.79,0.99], p = .0350). In Model 3, regular smokers (OR(regular smoker vs. others) = 45.00, CI: [9.98,202.94], p < .0001), and those who didn’t work (OR(No vs. full-time) = 4.45, CI: [1.16,17.02]), were more likely to live in families where smoking occurred in the home than did others. Ethnicity was also associated with smoking in the home (OR (Russia vs. Israel) = 4.03, CI: [0.93,17.37], OR(Other vs. Israel) = 0.41, CI [0.09,1.96], p = .0433). Risk perceptions were not significantly associated with family smoking practices in the home (p = .3224).

Model 4 is similar to Model 3, but does not include the variable for ethnicity. Most of the effects are of similar size and significance levels as in Model 3, with the exception of risk perception. The effect size of risk perception is similar in Models 2, 3, and 4 (Model 2: OR = .89, CI:[0.79,0.99], p = .0350; Model 3: OR = 0.93, CI: [0.81,1.07], p = .3224; Model 4: OR = .88, CI: [0.78,1.003], p = .0560), however, the p-value in Model 2 is statistically significant, in Model 4 is borderline significant, but in Model 3 is not statistically significant.

## Discussion

To the best of our knowledge, this is the first time that the recognized risk perception dimensions of likelihood, susceptibility, and severity [6] have been used to understand parental risk perceptions due to child tobacco smoke exposure. For the combined risk perception scale as well as for the three independent elements of likelihood, susceptibility, and severity, the relationship between risk perceptions and parental smoking status was clear: parents who were regular smokers assessed risk as significantly lower than did other participants (Univariate analysis: p = .0003, Multivariate analysis: p = .0158). The relationship between risk perceptions and family smoking in the home was less clear: higher assessments of risk were significantly associated with less family home smoking in the univariate model (p = .0350), but not the full multivariate model (p = .3224). Once ethnicity was removed from the full model, the significance decreased to borderline level (p = .0560). Particularly since ethnicity was significantly associated with risk perceptions (Model 1), it is possible that ethnicity acted as an effect modifier on the relationship between home exposure and risk perceptions, while controlling for smoking status.

Our finding of a clear association between PRETS and smoking status has strong support in the literature, with at least five previous studies with similar findings (Bock, [9] Chen, [10] Evans, [27] Lonergan, [12] and Lund [13]), and a single study with opposite findings [28].

Our finding that the association between PRETS and family smoking in the home was statistically significant in a simple analysis, but not in multivariate analyses, is similar to findings by Winickoff [16]: In his study, the relationship between secondhand smoke beliefs and a strict home smoking ban was significant in a bivariate but not multivariate analysis.

Evans and Gilmore [27] found that knowledge was associated with smoke-free homes, when using univariate analyses, and Lund [13] found an association between child exposure to tobacco smoke and health risk awareness using a univariate analytic approach. Helgason [29] found that health risk awareness was associated with TSE in the home in a multivariate analysis.

Based on our findings and on these studies, it seems that the association between PRETS and parental smoking may be somewhat more well-established than is the association between PRETS and smoking in the family home (and this relationship may be modified by ethnicity or other variables). This is somewhat counter-intuitive: PRETS is specifically about child exposure, not about parental smoking, and so one might assume that the more direct relationship between PRETS and family home smoking would be easier to establish. One possible explanation may be found in directionality of associations. Associations found in studies at a single point in time do not prove causality of effect in one direction or another [30]. In the case of PRETS and parental smoking, it is possible that the smoking behavior of the parent influenced his/her perceptions of risk of secondhand smoke for children, rather than that risk perceptions about child exposure influenced the decision of whether or not to smoke. This is especially true since the decision to start smoking was most likely made before the children were born. Another possible explanation is that while parental smoking status is unique to the parent, as are their perceptions, family smoking behavior in the home is a function of the behavior of that parent as well as others who live in the home. Even a parent convinced of harm due to tobacco smoke exposure may be unable to determine the behavior of others residing in the home. This could lead to a weaker association between PRETS and family smoking behavior. Finally, our findings suggest that there may be a more complex relationship between PRETS and family smoking in the home, which is modified by ethnicity.

### Measuring parental risk perceptions

Previous investigators have explored parental attitudes, knowledge, and risk perceptions regarding child tobacco smoke exposure. Questions asked by some previous authors are presented in Table 5, with a note on inferred dimension (likelihood, susceptibility, or severity; we added the possibility of a fourth category, “knowledge” to categorize questions which dealt with harm, without explicit reference to either likelihood, susceptibility, or severity). With the exception of Wagener, [23] investigators generally collected information on a single dimension. For example, Chen, [10] Drehmer, [22] McMillen, [17] and Winickoff [16] all asked about harm related to tobacco smoke exposure, using a question such as: “Inhaled smoke from a parent’s cigarette harms health of infants and children” [17]. Other investigators, for example, Evans, [27] Helgason, [29] Lonergan, [12] and Lund [13], asked about increased susceptibility to illness due to tobacco smoke exposure, with question such as: “Do you think that living with someone who smokes does, or does not, increase a child’s risk of asthma/ear infection/cot death/chest infections/other infections?” [27]. As in our study, susceptibility due to tobacco smoke exposure – not due to personal characteristics – was of interest. Bock [9] and Farber [11] asked about severity:”How much do you think other people’s smoking affects your baby’s health?” Wagener built constructs for precaution effectiveness, optimistic bias, and perceived vulnerability; his “optimistic bias” was closest to our susceptibility measure, while his perceived vulnerability included some questions about severity [23].

**Table 5:** **Questions from literature regarding risk perceptions and knowledge of child exposure to tobacco smoke**

**Study ID**	**Question + scale for answer**	**Author definition**	**Inferred dimension**
Bock	How much do you think other people’s smoking affects your baby’s health Scale: 1-5	Risk perception	Severity
Chen 2013	Smoking has bad impact on children’s health	Perception, consequence	Harm
	Children’s health is affected		
	Scale: 1–5, strongly agree to strongly disagree		
Drehmer, Winickoff	Breathing air in a room where people smoked yesterday can harm children today (third hand smoke)	Health risks (thirdhand smoke)	Harm
	Scale: 1–4 agreement, strongly agreed – strongly disagreed		
Evans and Gilmore	'Do you think that living with someone who smokes does, or does not, increase a child's risk of: asthma/ear infection/cot death/chest infections/other infections?'	Knowledge	Susceptibility
	Scale: Binary (yes/no)		
Farber	How much effect do you think exposure to tobacco smoke has on your child’s asthma?	Beliefs	Severity
	Scale: 4 categories: No/small/moderate/large negative effect.		
Helgason	Children exposed to ETS more likely to have inner ear/respiratory diseases/asthma attacks	Health risk awareness	Susceptibility
	Scale: 1-4		
Lonergan	A nonsmoker who regularly breathes in someone else’s smoke increases the risk of a nonsmoker getting… ear infections in children (Increases risk/does not increase risk)	Risk perceptions	Susceptibility
Lund & Helgason	Children exposed to ETS are more likely to have inner ear/respiratory diseases/	Health risk awareness	Susceptibility
	Scale: 1-4		
McMillan	Inhaled smoke from a parent’s cigarette harms health of infants and children	Knowledge of harm	Harm
	Scale: 1–4 agreement/ disagreement		
Wagener	From Perceived vulnerability scale:	Risk perception, Perceived vulnerability, optimism bias	Susceptibility
	How much do you believe that		
	a. your smoking is related to your child’s asthma symptoms		Severity
	b. your smoking increases the frequency of your child’s asthma attacks		Severity
	c. your smoking affects how bad your child’s asthma is		Severity
	d. your smoking increases the chance that your child will have to go to the emergency room or be hospitalized for an asthma attack?		Susceptibility/Severity
	From Optimistic Bias scale:		Susceptibility/Severity
	Compared to other children with asthma whose parents don’t smoke, what are the chances that		Susceptibility/Severity
	a. your child will have an asthma attack		Susceptibility/Severity
	b. your child’s asthma symptoms getting worse,		
	c. your child will have to visit the emergency room for an asthma attack		
	d. your child will have to visit a doctor because of worsening asthma		
	Scale: 1–5, low – high risk		
Winickoff 2009	Inhaled smoke from a parent’s cigarette harms health of infants and children	Health beliefs	Harm
			Harm
	Breathing air in a room today where people smoked yesterday can harm		
	Health of infants and children		
	Scale: 1–4 agreement		

As can be seen, there are currently no accepted standards for measurement of PRETS. Nor is it clear what the optimal way to measure PRETS should be, or how this should be determined. Two issues are of interest. First, measures with good predictive power would allow us identify populations subgroups which are likely to expose their children to tobacco smoke, and allow targeting of those groups for interventions. Second, the identification of dimensions of risk perception which differ between smoke-free and other homes could help craft messages to convince smokers to keep their homes smoke-free.

Some research has been done on comparing different types of measurement for risk perceptions and other psychosocial variables [8,21,26,31]. Baghal, in an article on measuring risk perceptions relating to smoking, [8] emphasizes that different measures may lead to different conclusions. He addressed verbal and probability scales, specifically addressing the relative benefits of asking about absolute risk, relative difference between smokers and nonsmokers, relative risk between smokers and nonsmokers, and vague quantifier scales, and concluded that “numeric measures are inconsistent with logical semantic understanding.” He also found that vague quantifier scales had better predictive power than did numeric scales.

Measures constructed from multiple questions are sometimes used in order to decrease the variability, or “noise” which would be present if a single question is measured. In addition to decreasing noise, there is an additional benefit to studying different dimensions of a perception: the answers regarding the individual dimension, and not just the constructed measure, may provide directions for successful interventions. For example, if those who allow their children to be exposed have relatively lower perceptions of severity of damage, then it might be worthwhile to address severity in the context of interventions, rather than susceptibility. On the other hand, if the key difference between those who expose and don’t expose their children is perceived susceptibility, the messages would better be focused on the additional risk of illness due to tobacco smoke exposure.

Our study showed that of the three components of the risk perception scale measured in the present study, the largest absolute difference between regular smokers and others in the mean risk perception value, and the smallest p-value, was for the severity component. Regarding family smoking in the home, the only dimension to reach statistical significant in a univariate analysis (controlled for smoking status) was susceptibility.

A review by Janz of the literature regarding the Health Belief Model showed that preventive health behaviors were associated with both susceptibility and severity. He found that perceived severity was weakly associated with preventive health behaviors, while perceived susceptibility was a relatively stronger contributor to preventive health behaviors [7].

Some evidence for the importance of severity in parental smoking behavior around children comes from a qualitative study of mothers of children with respiratory illnesses who smoked [32]. The authors found that some mothers disputed the severity of exposure to passive smoking, with one mother quoted as saying: “I don’t know if I believe it’s that bad for you. Not that bad” (p.108) Another mother was quoted as saying: “illness [was] not severe enough to warrant behavior change”; and another: “If he was really really severely asthmatic, I wouldn’t be smoking now.’ While far from conclusive evidence, these quotes suggest that underestimating the severity of harm due to TSE may be an important factor in parental smoking behavior around children. If true, this could provide a clue to creating effective interventions.

### Strengths and limitations

The strengths of this study include an innovative approach to measuring parental risk perceptions concerning child exposure to tobacco smoke, through the application of accepted psychological concepts, and the examination of the association between these risk perceptions and smoking status of respondent and home smoking behaviors. Careful pretesting of the survey instrument, and a very high response rate (nearly 98%), represent further strengths.

The cross-sectional nature of this study limited our ability to determine whether risk perceptions influenced behavior, as is postulated by the Health Belief Model, [5] or visa versa. As previously described [30], risk perception can influence behavior, and behavior can influence risk perception. Directionality can be assessed by longitudinal designs (experimental or observational), but not by a cross-sectional study such as this one.

The study population was not randomly sampled, however, concerns about possible selection bias are somewhat mitigated because people were recruited as they entered the clinic, and the response rate was nearly 98%. Participants were recruited from a single large clinic which represented a heterogeneous population. It is not possible to provide population level estimates for any variables, due to sampling of equal numbers of regular smokers and others. We did not explore risk perceptions due to third hand smoke exposure [16]. Our question on susceptibility related specifically to susceptibility due to tobacco smoke exposure, and not to innate, unique susceptibility of a particular child for reasons other than tobacco smoke exposure. Our findings suggested that ethnicity may be acting as an effect modifier on the relationship between family home smoking and risk perceptions. A larger sample size is necessary to further explore this relationship and to permit inclusion of more variables in the statistical model. Further study is needed to understand differences in perceptions between occasional, former, experimental, and never smokers, and explore these relationships in other populations.

## Conclusions

Parents who smoke regularly have lower risk perceptions regarding child tobacco smoke exposure than do others. An association between risk perceptions and home smoking behaviors is not proven and may be modified by ethnicity. At present, there are no standards for measurement of parental risk perceptions of child exposure to tobacco smoke. Identifying dimensions of risk perception which differ between smokers and nonsmokers, and between those parents who do and do not smoke around their children, may contribute to the creation of effective messages to promote child protection from tobacco smoke exposure.

## Abbreviations

TSE: Tobacco smoke exposure
PRETS: Parental Risk perceptions of Exposure to Tobacco Smoke
OR: Odds ratio
CI: Confidence interval
LSMeans: Least squared means

## References

[1] U.S. Department of Health and Human Services. The Health Consequences of Involuntary Smoking: A Report of the Surgeon General. Atlanta, Georgia 2006 [http://www.ncbi.nlm.nih.gov/books/NBK44324/]

[2] U.S. Department of Health and Human Services. The Health Consequences of Smoking - 50 Years of Progress: A Report of the Surgeon General. Altanta, Georgia 2014.

[3] Oberg M, Jaakkola MS, Woodward A, Peruga A, Pruss-Ustun A (2011). Worldwide burden of disease from exposure to second-hand smoke: a retrospective analysis of data from 192 countries. Lancet.

[4] Mannino DM, Caraballo R, Benowitz N, Repace J (2001). Predictors of cotinine levels in US children: data from the Third National Health and Nutrition Examination Survey. Chest.

[5] Rosenstock I (1974). Historical origins of the health belief model. Health Educ Monogr.

[6] Brewer NT, Chapman GB, Gibbons FX, Gerrard M, McCaul KD, Weinstein ND (2007). Meta-analysis of the relationship between risk perception and health behavior: the example of vaccination. Health Psychol.

[7] Janz N (1984). The Health Belief Model: A Decade Later. Health Educ Q.

[8] Baghal T (2011). The measurement of risk perceptions: the case of smoking. Journal of Risk Research.

[9] Bock B, Becker B, Borrelli B (2008). Smoking behavior and risk perception among parents of infants in the neonatal intensive care unit. Nicotine Tob Res.

[10] Chen YT, Hsiao FH, Miao NF, Chen PL (2013). Factors associated with parents perceptions of parental smoking in the presence of children and its consequences on children. Int J Environ Res Public Health.

[11] Farber H, Knowles S, Brown N, Caine L, Luna V, Qian Y (2008). Secondhand tobacco smoke in children with asthma: sources of and parental perceptions about exposure in children and parental readiness to change. Chest.

[12] Lonergan BJ, Meaney S, Perry IJ, Comber H, Power B, Bradley C (2014). Smokers still underestimate the risks posed by secondhand smoke: a repeated cross-sectional study. Nicotine Tob Res.

[13] Lund KE, Helgason AR (2005). Environmental tobacco smoke in Norwegian homes, 1995 and 2001: changes in children's exposure and parents attitudes and health risk awareness. Eur J Public Health.

[14] Robinson J, Kirkcaldy AJ (2007). ‘You think that I'm smoking and they're not’: why mothers still smoke in the home. Soc Sci Med.

[15] Rovira J, Viscusi W, Antonanzas F, Costa J, Hart W, Carvalho I (2000). Smoking risks in Spain: Part II.Perceptions of environmental tobacco smoke externalities. J Risk Uncertain.

[16] Winickoff JP, Friebely J, Tanski SE, Sherrod C, Matt GE, Hovell MF (2009). Beliefs about the health effects of "thirdhand" smoke and home smoking bans. Pediatrics.

[17] McMillen R, Winickoff J, Klein J, Weitzman M (2003). US adult attitudes and practices regarding smoking restrictions and child exposure to environmental tobacco smoke: changes in the social climate from 2000–2001. Pediatrics.

[18] Rosen L, Geva H, Connolly G (2011). Letter: political will ushers in a new era for tobacco control in Israel. Lancet.

[19] Johansson A, Halling A, Hermansson G, Ludvigsson J (2005). Assessment of smoking behaviors in the home and their influence on children's passive smoking: development of a questionnaire. Ann Epidemiol.

[20] Warren C, Riley L, Asma S, Eriksen M, Green L, Blanton C (2000). Tobacco use by youth: a surveillance report from the Global Youth Tobacco Survey project. Bull World Health Organ.

[21] Diefenbach M, Weinstein ND, O'Reilly J (1993). Scales for assessing health hazard susceptibility. Health Educ Res.

[22] Drehmer JE, Ossip DJ, Rigotti NA, Nabi-Burza E, Woo H, Wasserman RC (2012). Pediatrician interventions and thirdhand smoke beliefs of parents. Am J Prev Med.

[23] Wagener TL, Gregor KL, Busch AM, McQuaid EL, Borrelli B (2010). Risk perception in smokers with children with asthma. J Consult Clin Psychol.

[24] Kostjukovsky I (2010). Parental risk perceptions about infant exposure to secondhand smoke. Master’s Dissertation.

[25] Carifio J, Perla R (2007). Ten common misunderstandings, misconceptions, persistent myths and urban legends about Likert scales and Likert response formats and their antidotes. Journal of Social Sciences.

[26] Eibner F, Barth J, Helmes A, Bengel J (2006). Variations in subjective breast cancer risk estimations when using different measurements for assessing breat cancer risk perception Health. Risk & Society.

[27] Evans K, Sims M, Judge K, Gilmore A (2011). Assessing the knowledge of the potential harm to others caused by second-hand smoke and its impact on protective behaviors at home. J Public Health.

[28] Shiva F, Padyab M (2008). Smoking practices and risk awareness in parents regarding passive smoke exposure of their preschool children: a cross-sectional study in Tehran. Indian J Med Sci.

[29] Helgason AR, Lund KE (2001). Environmental tobacco smoke exposure of young children–attitudes and health-risk awareness in the Nordic countries. Nicotine Tob Res.

[30] Brewer NT, Weinstein ND, Cuite CL, Herrington JE (2004). Risk perceptions and their relation to risk behavior. Ann Behav Med.

[31] Klein W, Stefanek M (2007). Cancer risk elicitation and communication: lessons from the psychology of risk perception. CA Cancer J Clin.

[32] Coxhead L, Rhodes T (2006). Accounting for risk and responsibility associated with smoking among mothers of children with respiratory illness. Sociol Health Illn.

